# Neuralgia as a "Warning"

**Published:** 1881-08

**Authors:** 


### NEURALGIA AS A "WARNING."

The great prevalence of "neuralgia"—or what commonly goes by that name—should be regarded as a warning indicative of a low condition of health, which must necessarily render those who are affected with this painful malady especially susceptible to the invasion of diseases of an aggressive type. This is the season at which it is particularly desirable to be strong and well furnished with the sort of strength that affords a natural protection against disease. There will presently be need of all the internal heat which the organism can command, and a good store of fat for use as fuel is not to be despised. It is no less essential that the vital forces should be vigorous, and the nerve power, especially in full development. Neuralgia indicates a low or depressed state of vitality, and nothing so rapidly exhausts the system as pain that prevents sleep and agonizes both body and mind. It is, therefore, of the first moment that attacks of this affection, incidental to and indicative of a poor and weak state, should be promptly placed under treatment, and as rapidly as may be controlled. It is worth while to note this fact, because, while the spirit of manliness incites the "strong-minded" to patient endurance of suffering, it is not wise to suffer the distress caused by this malady, as many are now suffering it, without seeking relief, forgetful of the condition it bespeaks, and the constitutional danger of which it is a warning sign.—*Lancet.*

